# Asymmetric and Symmetric Dimethylarginine as Risk Markers for Total Mortality and Cardiovascular Outcomes: A Systematic Review and Meta-Analysis of Prospective Studies

**DOI:** 10.1371/journal.pone.0165811

**Published:** 2016-11-03

**Authors:** Sabrina Schlesinger, Svenja R. Sonntag, Wolfgang Lieb, Renke Maas

**Affiliations:** 1 Institute of Epidemiology, Christian-Albrechts University of Kiel, Kiel, Germany; 2 Institute of Experimental and Clinical Pharmacology and Toxicology, Friedrich-Alexander-Universität Erlangen-Nürnberg, Erlangen, Germany; The University of Tokyo, JAPAN

## Abstract

**Background:**

A growing number of studies linked elevated concentrations of circulating asymmetric (ADMA) and symmetric (SDMA) dimethylarginine to mortality and cardiovascular disease (CVD) events. To summarize the evidence, we conducted a systematic review and quantified associations of ADMA and SDMA with the risks of all-cause mortality and incident CVD in meta-analyses accounting for different populations and methodological approaches of the studies.

**Methods:**

Relevant studies were identified in PubMed until February 2015. We used random effect models to obtain summary relative risks (RR) and 95% confidence intervals (95%CIs), comparing top versus bottom tertiles. Dose-response relations were assessed by restricted cubic spline regression models and potential non-linearity was evaluated using a likelihood ratio test. Heterogeneity between subgroups was assessed by meta-regression analysis.

**Results:**

For ADMA, 34 studies (total n = 32,428) investigating associations with all-cause mortality (events = 5,035) and 30 studies (total n = 30,624) investigating the association with incident CVD (events = 3,396) were included. The summary RRs (95%CI) for all-cause mortality were 1.52 (1.37–1.68) and for CVD 1.33 (1.22–1.45), comparing high versus low ADMA concentrations. Slight differences were observed across study populations and methodological approaches, with the strongest association of ADMA being reported with all-cause mortality in critically ill patients. For SDMA, 17 studies (total n = 18,163) were included for all-cause mortality (events = 2,903), and 13 studies (total n = 16,807) for CVD (events = 1,534). High vs. low levels of SDMA, were associated with increased risk of all-cause mortality [summary RR (95%CI): 1.31 (1.18–1.46)] and CVD [summary RR (95%CI): 1.36 (1.10–1.68) Strongest associations were observed in general population samples.

**Conclusions:**

The dimethylarginines ADMA and SDMA are independent risk markers for all-cause mortality and CVD across different populations and methodological approaches.

## Introduction

Asymmetric (ADMA) and symmetric (SDMA) dimethylarginine are both di-methylargingines that are structurally related to L-Arginine and, therefore, may interfere with L-Arginine-related signaling. Both share transport mechanisms of L-Arginine,[1] but likely differ in their functional effects on nitric oxide (NO) synthesis. Whereas ADMA is an established competitive inhibitor of the NO Synthasis,[2] SDMA has no or only little effect on NO-synthesis.[3] Particularly for ADMA, experimental and clinical data support a role in vascular remodeling,[4] [5] e.g. by demonstrating that vascular function and the degree of atherosclerosis correlated with ADMA levels in animal models.[5] On a parallel note, administration of ADMA in humans lead to reduced cardiac output and renal plasma flow and to increased vascular resistance.[6] Due to their biological functions, both markers have been explored as cardiovascular biomarkers.

Since the landmark studies by Zocalli et al.[7] and Valokonen et al.[8] in 2001, multiple studies have linked circulating ADMA concentrations to cardiovascular disease (CVD) risk and mortality and many reported positive associations.[9–11] However some conflicting results were observed; for example in the Framingham Offspring Study, ADMA was positively related to all-cause mortality, but not to incident CVD.[12] With respect to SDMA, initial studies did not find an association with adverse outcomes,[7, 13, 14] but some more recent studies reported positive associations with all-cause mortality or CVD.[11, 15, 16] Overall, prior studies on ADMA/SDMA and CVD or all-cause mortality were derived from many different study populations (general population,[15, 17, 18] patients with CVD,[9, 11, 19, 20] renal diseases,[7, 13, 21–24] diabetes,[25, 26] or critically ill patients from intensive care unit,[27–29] respectively), differed in their methodological approaches (e.g. using types of samples (plasma vs. serum) or methods to determine the biomarker levels (HPLC vs. tandem mass spectroscopy vs. ELISA), or considered different confounders in their analysis.

A recent meta-analysis strengthened the notion that ADMA is an independent risk marker for cardiovascular events but could not confirm an association of SDMA with incident CVD.[30] While these data were intriguing, the exact shape of the dose-response relation between these dimethylarginines and CVD has not been described. In addition, the mentioned meta-analysis focused on ADMA/SDMA and CVD, but did not relate the biomarkers to all-cause mortality. Thus, we aim to quantify associations of ADMA and SDMA level with all-cause mortality and CVD in a systematic review and meta-analyses by accounting for differences in study populations (including participants from the general population as well as individuals with underlying diseases) and methodological approaches of the underlying studies.

## Methods

This report follows the Preferred Reporting Items for Systematic Reviews and Meta-Analyses (PRISMA) statement, [31] and the complete PRISMA checklist is provided in S1 Fig.

### Search strategy

A systematic literature search was performed in MEDLINE (PubMed) by two independent investigators (S.S. and R.M.) to identify prospective studies, published until February 2015 that examined the relation between ADMA and SDMA (as exposure variables, each exposure considered separately) and all-cause mortality and CVD (as outcome, each outcome considered separately). There were no limits used in the searches.

Search terms included (ADMA OR dimethylarginin* OR SDMA) AND ("myocardial infarction" OR death OR mortality OR stroke OR "major adverse cardiac events" OR cardiovascular OR CAD OR "Coronary artery disease" OR CVD OR "Cardiovascular disease" OR "coronary heart disease" OR CHD) AND (Follow-up OR "clinical trial" OR Longitudinal OR prospective OR nested OR cohort OR observational OR endpoint OR "cox regression" OR outcome OR survival OR predict*). Furthermore, reference lists of relevant papers and previous reviews were hand-searched to assess additional potentially relevant articles.

### Data selection and extraction

#### Inclusion criteria

We included studies if 1) studies had investigated the association between ADMA and/or SDMA with risk of all-cause mortality and/or CVD, 2) studies were original articles, 3) studies had a prospective design, 4) the associations was presented as odds ratio (OR), relative risk (RR) or hazard ratio (HR). In the present manuscript RR stands for all three estimates.

Studies were excluded if 1) they were not reported in English language, 2) they focused on a mixed endpoint (combined endpoint e. g. major adverse cardiac event (MACE), including all-cause mortality and CVD), and 3) studies did not report how ADMA and SDMA were modeled in the statistical analyses (e. g. if it was not clear whether they were modeled as categorical or continuous traits; or when it was unclear whether effect estimates were given per 1-unit or per 1-SD increment in biomarker levels). If such relevant information was missing in the publication, we contacted the corresponding author, and if authors responded, studies were included in our meta-analysis.[10, 16, 29, 32–34]

In the case that multiple studies reported on one dataset, we included the most recent study in our meta-analysis.

Furthermore, three investigators (S.S., R.M. and S.R.S.) independently extracted for each study, information about the first author, publication year, country of the study, study source and design, duration of follow-up, characteristics of the study sample (general population or patients with specific diseases), sex, age, sample size, outcome, type of estimate, number of cases, exposure assessment and categorization, as well as the most comprehensively adjusted OR, RR or HR, with the respective adjustment variables. Estimates and their 95% confidence intervals (95% CI) for ADMA/SDMA in relation to all-cause mortality or CVD were extracted as they were presented in the original reports, including estimates per 1-unit increments, per 1-SD increment, or for the highest versus the lowest categories (e.g. tertiles or quartiles) of ADMA or SDMA, respectively. We contacted the corresponding author, if relevant information was missing.

#### Definition of outcomes

We focused on two outcomes: 1) all-cause mortality and 2) CVD, which was defined as fatal and nonfatal CVD and coronary heart disease events, as it has been done in previous work.[35] Studies that provided effect estimates for a combined outcome, including all-cause mortality and CVD, were not included in our meta-analysis. If studies reported findings for defined sub-outcomes (e.g. cause-specific mortality) or sub-groups of the overall sample (e.g. stratified for patients with and without surgery), we combined theses sub-group/sub-outcome results to estimate effect estimates for the overall sample,[9, 36–38] by using fixed effect models.

### Statistical analyses

We conducted four main analyses: we investigated the associations between 1) ADMA levels and all-cause-mortality, 2) ADMA levels and incident CVD, 3) SDMA levels and all-cause mortality, and 4) SDMA levels and incident CVD.

#### High versus low biomarker level

In the individual studies the exposure variable (the biomarker concentrations) were modeled in different ways (either as a continuous or as a categorical trait) and the effect measures (HRs/RRs/ORs with their corresponding 95% CIs) were reported per different increments in the exposure variable (e.g. per 0.1 μmol/L, per 1 μmol/L or per 1-SD increment in the continuous biomarker; or per tertiles, quartiles or quintiles in biomarker levels; or comparing individuals above vs. below the median). In the present meta-analysis, we harmonized the presentation of the data by providing effect measures for the top vs. the bottom tertile of the ADMA or SDMA distribution. If the effect measures in the original publication were not presented per tertiles, the results had to be converted to a standard scale of effect, by giving an estimate per 2.18 SD units of ADMA or SDMA as described by Danesh *et al*.[39] The factor of 2.18 is the difference in the means of the upper and lower tertile of the standard normal distribution. Thus, this scaling method assumes that the exposure (ADMA or SDMA) follows a normal distribution and the association with disease risk (CVD or mortality) is log-linear. If the original publication reported effect measures by biomarker quartiles, the effect measure of the top vs. the bottom quartile was log-transformed, then multiplied by the factor 2.18/2.54 and the resulting product was exponentiated to the base of e to derived a RR comparing the top vs. the bottom tertile.[39] For studies reporting results by biomarker quintiles or comparing individuals above vs. below the median we used scaling factors of 2.18/2.80 and 2.18/1.59, respectively.[39] If the original studies reported relative risk increases per 1-SD increment in ADMA or SDMA levels, the log risk ratio was multiplied by 2.18 and subsequently presented as effect measure of the top vs. bottom tertile. Risk ratio that were reported per 1-unit increment, were multiplied by the study specific SD. If some individual studies did not report the study specific biomarker SD for their sample, we used the SD of the largest study (0.14 μmol/L for ADMA and 0.12 μmol/L for SDMA; Email communication with the corresponding author of that study).[16]

Summary RRs (95% CIs) for ADMA and SDMA (each biomarker considered separately) and all-cause mortality and/or CVD (each outcome considered separately) were calculated by applying random effect models. Heterogeneity between studies was evaluated using I^2^ statistics.

#### Subgroup analyses

To assess for risk of bias, we conducted subgroup analyses considering the following factors:

- underlying study population (general population, patients with renal disease, patients with prevalent CVD, patients with diabetes mellitus, and, for the analyses with respect to all-cause mortality, critically ill patients from intensive care units),
- number of events (<100, 100 -<200 and ≥200),
- duration of follow-up (longer or shorter than the mean across all studies: <4.7 years and ≥4.7 years),
- type of blood sample (plasma and serum) used for analyses,
- method for ADMA/SDMA measurement (HPLC, tandem mass spectrometry and ELISA), and
- adjustment for number of important confounders (0–2, 3–5 and ≥6, whereas important confounders included age, sex, BMI/or waist circumference, smoking, history of CVD, diabetes, blood pressure/hypertension, blood lipids/hyperlipidemia, family history of CVD and GFR/eGRF).

Heterogeneity between subgroups was assessed by applying meta-regression analysis.

#### Dose response meta-analysis

In a dose-response meta-analysis, we investigated ADMA and SDMA as continuous traits (effect measures per 0.1 μmol/L in biomarker levels) in relation to all-cause mortality and CVD, respectively. If the original publication provided estimates per 1 μmol/L increment in biomarker levels, these were converted into estimates per 0.1 μmol/L increment. If studies did not report the association with all-cause mortality, or CVD with ADMA or SDMA being modeled as a continuous trait but rather for categories of the biomarker, we used the method described by Orsini *et al*. and by Greenland and Longnecker.[40, 41] Here, the study specific slopes and 95% CIs were estimated from the natural logarithms of the RRs and 95% CIs across the categories of ADMA or SDMA, respectively. This requires from each individual study the RR (with 95% CI), the quantified exposure value, and the number of cases and person years for at least three exposure categories.

For studies that did not report the number of person-years in the individual exposure categories, we estimated the person-years per category based on the number of cases per category and the total person-years/ total number of participants and the follow-up period of the entire study, as suggested previously.[42, 43]

For any missing information, we contacted the corresponding author.[15, 16, 44–46] If missing information could not be obtained, we excluded the respective studies from the dose-response analysis.[15, 45, 46] If the author reported the range (rather than the mean) to define an exposure category, we estimated the mean value as previously described.[47, 48]

Finally, we performed cubic spline regression models to explore a potential non-linear relation between di-methylarginines (ADMA/ SDMA) and all-cause mortality or CVD, respectively. For this specific analysis only studies with information on at least three exposure categories (please see above) could be included. To formally test for non-linearity, we used a likelihood ratio test.[49] In a sensitivity analysis, we restricted our analysis to studies which determined ADMA/SDMA by tandem mass spectrometry and ELISA because these methods allowed more reliable quantification of the absolute concentration as compared to the assessment by some of the early HPLC methods (predominantly used in older studies).

All analyses were performed using SAS version 9.3 (SAS Institute, Inc., Cary, NC, USA), R statistical software version 3.0.3 (R Foundation for Statistical Computing, Vienna, Austria) and the R package meta version 3.6–0 (Schwarzer, Freiburg, Germany) and metafor version 1.9–3 (Viechtbauer, Maastricht, The Netherlands). A two-tailed p-value of <0.05 was considered as statistical significant.

## Results

### Literature search and study characteristics

Overall, we identified 711 studies, from which titles and abstracts were reviewed. After screening full-texts of 102 studies, we finally included 50 studies in our meta-analysis (S2 Fig).

Out of these, 34 studies investigated the association between ADMA and all-cause mortality, 30 studies evaluated the association between ADMA and CVD, and 17 studies examined the relation between SDMA and all-cause. A total of 13 studies assessed the association of SDMA with CVD. Across all studies, the mean follow-up time was 4.7 years (range: 0.1–11.3 years).

Characteristics of the included studies are provided in Table 1 and S1 Table. A total of 8 studies were based on participants of the general population, 12 studies included patients with renal disease, 23 studies included individuals with prevalent CVD, 4 studies provided results for individuals with diabetes mellitus, and 4 studies included patients from the intensive care unit. ADMA and SDMA were measured in plasma in 35 and in serum in 15 studies. In addition, ADMA and SDMA were analyzed using HPLC in 27 studies, using tandem mass spectrometry in 13 studies and with ELISA in 10 studies. The confounders included in the individual studies are shown in the S1 Table.

**Table 1:** Characteristics of studies included in the meta-analysis focusing on ADMA/SDMA and/or all-cause mortality/CVD.

First author, year (country)	Study source, study design	Duration of follow-up in y	Study population	Sex, Mean age in y	Sample size	Outcome	Type of estimate	No. of events of each outcome	Method for ADMA measurements, Type of sample (Plasma/ Serum)	ADMA categories	SDMA categories
Valkonen et al., 2001 (Finland)[8]	Kuopio Ischemic Heart Disease Risk Factor Study; prospective nested case-control study	7 y	Middle-aged men with and without acute coronary events in the past and who did not smoke	M, 59.9 y	150	CVD (fatal and nonfatal CV events)	OR	45	HPLC, serum	Quartiles: highest (>0.62 μmol/L) Per 0.1 μmol/L	-
Zoccali et al., 2001 (Italy)[7] (only included for SDMA; for ADMA: most recent report by Tripepi, 2011)	Prospective cohort study	2.8 y	Patients with end-stage renal disease with haemodialysis	M & F, 59.9 y	225	All-cause mortality CVD (fatal and nonfatal CV events)	HR	83 81	HPLC, plasma	Quartiles: highest (>3.85 μmol/L, with quartile 1+2 as reference) Per 1 μmol/L	Per 1 μmol/L
Lu et al., 2003 (China)[50]	Prospective cohort study	1.3 y	Patients with stable angina and undergoing percutaneous coronary intervention	M & F, 71 y	153	CVD (fatal and nonfatal CV events)	RR	51	HPLC, plasma	Tertiles: highest 0.62 μmol/L Per 0.1 μmol/L	Per 1 μmol/L
Nijveldt et al., 2003 (The Netherlands)[27]	Prospective cohort study	na	Critical ill patients on the surgical intensive care unit	M & F, 58 y	52	All-cause mortality	OR	21	HPLC, plasma	Quartiles: highest (>1.05 μmol/L) Per 1 μmol/L	Quartiles: highest (na)
Ravani et al., 2005 (Italy)[51]	Prospective cohort study	2.3 y	Patients with chronic kidney disease (end-stage renal disease)	M & F, 71 y	131	All-cause mortality	HR	31	ELISA, plasma	Per 0.1 μmol/L	-
Schnabel et al., 2005 (Germany)[9]	AtheroGene Study, prospective cohort study	2.6 y	Patients with coronary artery disease	M & F, 61 y	1874	All-cause mortality CVD (death from cardiovascular cause or nonfatal MI)	HR	101 114	ELISA, serum	Tertiles: highest (>0.7 μmol/L) Per 1 SD	-
Busch et al., 2006 (Germany)[52]	Prospective cohort study	2 y	Patients with chronic kidney disease	M & F, 57.6 y	200	CVD	HR	47	HPLC, plasma	Quartiles: highest >1.17 μmol/L	Quartiles: highest (>2.82 μmol/L) -
Mittermayer et al., 2006 (Austria)[45]	Prospective cohort study	1.6 y	Patients with advanced peripheral artery disease	M & F, 70 y	496	All-cause mortality	HR	64	HPLC, plasma	Quartiles: highest ≥0.64 μmol/L	-
Maas et al., 2007 (Germany)[53]	KORA; prospective nested case-control study	6.2 y	Initially healthy men	M, 61.2 y	342	CVD	HR	88	ELISA, plasma	Tertiles: highest ≥0.86 μmol/L	-
Nicholls et al., 2007 (US)[54]	SHOCK-2 trial and Genebank study; prospective cohort study	0.1 y	Patients with cardiogenic shock after acute MI	M & F, 72 y	79	All-cause mortality	OR	31	Tandem mass spectrometry, plasma	Median: higher ≥1.25 μmol/L	-
Skoro-Sajer et al., 2007 (Austria)[36]	Prospective study	3.1 y	Patients with chronic thrombo-embolic pulmonary hypertension without unstable atherosclerotic vascular disease, renal dysfunction, untreated hyperlipidemia, obesity and smoking	M & F, 59 y	135	All-cause mortality	HR	53	HPLC, plasma	Cut-off by ≥0.64 μmol/L	-
Lajer et al., 2008 (Denmark)[25]	Steno Diabtes Center, prospective cohort study	11.3 y	Patients with type 1 diabetes	M & F, 42.1 y	397	All-cause mortality; CVD (fatal and nonfatal CV events)	HR	126 116	HPLC, plasma	Median: higher: ≥0.46 μmol/L	-
Leong et al, 2008 (Sweden)[55]	Population Study of Women in Gothenburg, prospective cohort study	24 y	Healthy population-based women	F, 46 y	880	All-cause mortality CVD (fatal and nonfatal CV events)	RR	138 101	HPLC, plasma	Per 1 SD (0.15 μmol/L)	-
Wilson Tang et al., 2008 (US)[56]	ADEPT-study; prospective cohort study	2.8 y	Patients with chronic systolic heart failure	M & F, 57.8 y	132	All-cause mortality	HR	20	Tandem mass spectrometry, Plasma	Per 1 SD (0.14 μmol/L)	-
Zeller et al., 2008. (France)[14]	Prospective cohort study	1 y	Patients with acute MI	M & F, 68.7 y	249	All-cause mortality CVD (fatal CV events; only for ADMA)	HR	34 31	HPLC, serum	Tertiles: highest ≥1.14 μmol/L	Per 1 μmol/L
Aucella F et al., 2009 (Italy)[13]	Prospective cohort study	4.6 y	Patients with end stage renal disease	M & F, 58 y	288	All-cause mortality CVD (fatal CV events)	HR	140; 70	HPLC, Plasma	Per 1 μmol/L	Per 1 μmol/L
Böger et al., 2009 (US)[18]	Framingham Offspring Study; prospective cohort study	10.9 y	Population based free of CVD at baseline	M & F, 59 y	3,320	All-cause mortality CVD (fatal and nonfatal CV events)	HR	285 281	Tandem mass spectrometry, Plasma	Quartiles highest value not reported Per 1 SD (0.13 μmol/L)	-
Cavusoglu et al., 2009 (US)[57]	Veterans Affairs Medical Center, prospective cohort study	2 y	Patients with acute coronary syndrome	M, 64.8 y	182	All-cause mortality	HR	26	ELISA, serum	Tertiles: highest ≥1.115 μmol/L (t1+t2 = ref)	-
Kiechl et al., 2009 (Italy)[58]	Bruneck study, prospective cohort study	5 y	General population	M & F, 66.2 y	572	CVD (fatal and nonfatal CV events, stroke)	HR	43	Tandem mass spectrometry, plasma	Per 1 μmol/L	Quartiles: highest ≥0.80 μmol/L; per 1 μmol/L
Wang et al., 2009 (US)[59]	Cleveland Clinic GENEBANK study; prospective cohort study	3 y	Patients with and without significantly obstructive CAD	M & F, 63.9 y	955	All-cause mortality	HR	131	Tandem mass spectrometry, plasma	Quartiles: highest ≥1.49 μmol/L	Quartiles: highest ≥1.05 μmol/L
Young et al., 2009 (US)[21]	The Modification of Diet in Renal Disease Study, randomized controlled trial	9.5 y	Patients with stage 3 to 4 chronic kidney disease	M & F, 52 y	820	All-cause mortality CVD (fatal CV events)	HR	202 122	ELISA, serum	Per 1 SD (0.25 μmol/L)	-
Abedini et al., 2010 (Norway)[22]	ALERT-study; randomized, double-blind, placebo-controlled study	6.7 y	Patients with renal transplant with stable graft function	M & F, 49.7 y	1,847	All-cause mortality CVD (fatal and nonfatal CV events)	HR	343 207	HPLC, serum	Quartiles highest ≥0.86 μmol/L	-
Ari et al., 2010 (Turkey)[60]	Prospective cohort study	0.5 y	Patients with elective percutaneous transluminal coronary angioplasty and stent	M & F, 57.3 y	92	CVD (fatal and nonfatal CV events, recurrent revascularization)	HR	36	ELISA, serum	Per 1 μmol/L	-
Cavusoglu et al. 2010 (US)[26]	Veterans Administration Medical Center; prospective cohort study	2 y	Patients with diabetes mellitus	M, 65.9 y	162	All-cause mortality	HR	24	ELISA, plasma	Tertiles: highest ≥1.05 μmol/L (t2+t1 = ref)	-
Schulze et al., 2010 (UK)[46]	Prospective cohort study	4.3 y	Patients with acute ischemic stroke (survived first 30 days after acute stroke)	M & F, 69.8 y	394	All-cause mortality	HR	231	Tandem mass spectrometry, plasma	Quartiles: highest >0.50 μmol/L	Quartiles: highest >0.48 μmol/L
Shi et al., 2010 (China)[61]	Prospective cohort study	1.3 y	Patients with chronic kidney disease and healthy controls	M & F, 45.6 y	91	CVD (fatal and nonfatal CV events)	HR	25	HPLC, plasma	By median	-
Yeo, et al., 2010 (Indonesia)[62]	Prospective cohort study	na	Patients with severe malaria	M & F, 29 y	49	All-cause mortality	OR	8	HPLC, plasma	Per 1 μmol/L	Per 1 μmol/L
Böger, et al., 2011 (Germany)[10]	GetABI cohort, prospective cohort study	5 y	Primary care patients; with and without peripheral arterial disease	M & F, 73.1 y	2,447	All-cause mortality CVD (fatal and nonfatal CV events)	HR	390 296	Tandem mass spectrometry, plasma	Quartiles: highest >0.70 μmol/L	Quartiles: highest >0.57 μmol/L
Davis et al., 2011 (Australia)[28]	Prospective cohort study	0.1 y	Patients with sepsis and controls	M & F, 50.5 y	98	All-cause mortality	OR	6	HPLC, Plasma	Quartiles: highest ≥0.66 μmol/L	Quartiles: highest ≥1.30μmol/L
Lu et al., 2011b (China)[63]	Prospective cohort study	2.4 y	Individuals referred for coronary angiography (patients with and without CAD)	M & F, 66.9 y	997	All-cause mortality CVD (fatal and nonfatal CV events, stroke)	HR	64 81	HPLC, Plasma	Tertiles: highest >0.48 μmol/L Per 0.1 μmol/L	-
Meinitzer et al., 2011 (Germany)[11]	LURIC study, prospective cohort study	7.7 y	Individuals referred for coronary angiography (patients with and without CAD)	M & F, 62,6 y	3,229	All-cause mortality CVD (fatal CV events)	HR	749 469	HPLC, Serum	Quartiles: highest >0.89 μmol/L	Quartiles: highest >0.63 μmol/L
Tripepi et al., 2011 (Italy)[64]	Cardiovascular Risk Extended Evaluation in Dialysis Patients cohort, prospective cohort study	13 y	Patients with end stage renal disease	M & F, 60 y	225	All-cause mortality CVD (fatal and nonfatal CV events)	HR	160 123	HPLC, plasma	Per 1 μmol/L	-
Anderssohn et al., 2012 (Germany)[37]	Prospective cohort study	3.3 y	Patients with chronic heart failure: dilated cardiomyopathy and ischemic cardiomyopathy	M & F, 55.1 y	341	All-cause mortality	HR	101	Tandem mass spectrometry, plasma	Log, per 1 μmol/L	Quartiles: highest >0.50 μmol/L Log, per 1 μmol/L
Borgeraas et al., 2012 (Norway)[19]	BECAC, prospective cohort study	5.3 y	Patients with suspected coronary artery disease	M & F, 61.0 y	1,364	CVD (fatal and nonfatal CV events)	HR	129	Tandem mass spectrometry, plasma	Quartiles: highest ≥0.70 μmol/L	-
Cavalca et al., 2012 (Italy)[65]	Prospective cohort study	1.8 y	Patients with acute coronary syndrome/ consecutive patients with Non-ST-elevation myocardial infarction	M & F, 66.5 y	104	CVD (CV events and reinfarction)	HR	24	HPLC, plasma	Median: >0.42 μmol/L	Median: >0.46 μmol/L
Hsu et al., 2012 (China)[66]	Prospective cohort study	2.4 y	Patients with ischemic chronic heart failure	M & F, 70 y	285	CVD (fatal and nonfatal CV events, stroke)	HR	58	HPLC, plasma	Best predictive value: >0.48 μmol/L Per 1 SD (0.12 μmol/L)	-
Visser et al., 2012 (The Netherland)[29]	Prospective cohort study	na	Patients with septic or cardiogenic schock	M & F, 65.7 y	44	All-cause mortality	OR	16	HPLC, plasma	Per 1 μmol/L	-
Zairis et al., 2012 (Greece)[67]	Prospective cohort study	1 y	Patients with acute decompensation of chronic heart failure and reduced left ventricular ejection fraction	M & F, 73 y	651	CVD (fatal CV events)	HR	237	HPLC, plasma	Quartiles: highest ≥1.83 μmol/L	-
Gore et al., 2013 (US)[15]	Dallas Heart Study (DHS), prospective cohort study	7.4 y	General population	M & F, 43 y	3.411	All-cause mortality CVD (fatal CV events)	HR	161 62	Tandem mass spectrometry, plasma	Quintiles: highest ≥0.58 μmol/L Per log unit change	Quintiles: highest ≥0.50 μmol/Lper log unit change
Ignjatovic et al., 2013 (Serbia)[68]	Prospective cohort study	3 y	Patients with end-stage renal disease in hemodialysis	M & F, 58.0 y	153	All-cause mortality CVD (fatal CV events)	HR	61 37	HPLC, plasma	Per 1 μmol/L	Per 1 μmol/L
Koch et al., 2013a (Germany)[32]	Prospective cohort study	~3 y	Patients with critically illness; with and without sepsis	M & F, 63 y	247	All-cause mortality	HR	115	ELISA, serum	-	Per 1 μmol/L
Koch et al., 2013b (Germany)[34]	Prospective cohort study	3 y	Patients with critically illness; with and without sepsis	M & F, 63 y	255	All-cause mortality	HR	120	ELISA, serum	Per 1 μmol/L	-
Pizzarelli et al., 2013 (Italy)[69]	InCHIANTI study; prospective cohort study	9.2 y	General population aged ≥ 65 years	M & F, 75 y	1,025	All-cause mortality CVD (fatal CV events)	HR	384 141	Tandem mass spectrometry, plasma	Per 0.1 μmol/L	-
Siegerink et al., 2013 (Germany)[20]	KAROLA study; prospective cohort study	8.1 y	Patients with stable coronary heart disease	M & F, 58.7 y	1,148	All-cause mortality secondary CVD (fatal and nonfatal CV events)	HR	121 150	Tandem mass spectrometry, plasma	Quartiles: highest ≥0.64 μmol/L Per 1 SD (0.12 μmol/L)	Quartiles: highest ≥0.59 μmol/L Per 1 SD (0.16 μmol/L)
Drew et al., 2014 (US)[44]	Cognition and Dialysis study cohort; prospective cohort study	2.3 y	Patients on hemodialysis	M & F, 63 y	259	All-cause mortality	HR	130	HPLC, plasma	Quartiles: highest mean 0.98 μmol/L Per 1 SD (0.15 μmol/L)	-
Levin et al., 2014 (Canada)[23]	Can-PREDDICT; prospective cohort study	1 y	Patients with chronic kidney disease	M & F, 68 y	2,402	All-cause mortality	HR	137	ELISA, serum	Per 1 SD (0.11 μmol/L)	-
PihlstrØm et al., 2014 (Europe, Canada)[24]	ALERTstudy; prospective cohort study	5.1 y	Renal transplant recipients	M & F, 49.7 y	925	All-cause mortality CVD (fatal CV events)	HR	125 65	HPLC, serum	-	Quartiles: highest ≥1.38 μmol/L
Plicner et al., 2014 (Poland)[38]	Prospective cohort study	na	Patients following coronary artery bypass grafting	M & F, 65.2 y	158	CVD (fatal CV events)	OR	19	HPLC, plasma	Per 1 μmol/L	-
Schwedhelm et al., 2014 (Germany)[16]	SHIP; prospective cohort study	10.1 y	General population	M & F, 51 y	3,952	All-cause mortality; CVD (fatal CV events)	HR	426 139	Tandem mass spectrometry, serum	Tertiles: highest ≥0.72 μmol/L Per 1 SD (0.14 μmol/L)	Tertiles: highest ≥0.49 μmol/L; Per 1 SD (0.12 μmol/L)
Yilmaz et al., 2014 (Turkey)[70]	Prospective cohort study	3,3 y	Patients with familial Mediterranean fever-related amyloidosis or primary glomerulopathies	M & F, 32 y	200	CVD (fatal and nonfatal CV events)	HR	54	HPLC, serum	Per 1 μmol/L	-

-, not measured; CVD, cardiovascular disease; na, not available; sd, standard deviation; y, years

### Association between ADMA and all-cause mortality

**Fig 1:**
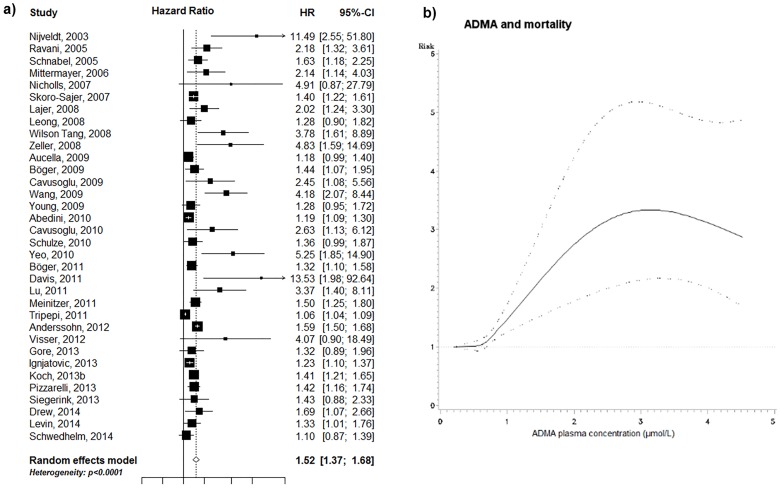
ADMA and all-cause mortality: a) high versus low analysis, and b) non-linear dose-response analysis (based on 7 studies, p for non-linearity = 0.035). Black square: point estimate for individual study; horizontal line: 95% CI for observed effect in each study; diamond: pooled estimate and 95% CI for meta-analysis. Random-effects estimate (DerSimonian and Laird method).

Across all studies (n = 34, involving 32,428 subjects of which 5,035 died; mean follow-up: 5.4 years), higher ADMA levels were associated with greater risk for all-cause mortality (summary RR: 1.52; 95% CI, 1.37–1.68, p<0.0001; comparing participants in the top ADMA tertile with participants in the bottom ADMA tertile; Fig 1a). There was evidence for statistical significant heterogeneity across studies (I² = 88.9%, p<0.0001).

In subgroup analyses, the strongest association was reported in critically ill patients from intensive care units [summary RR: 4.85; 95% CI, 1.39–16.97, p = 0.014; based on 4 studies (p_heterogenity_<0.001); Table 2]. However, the association between ADMA and all-cause mortality was also statically significant in the general population, and in patients with renal diseases and in those with prevalent CVD. The association in patients with diabetes mellitus reached borderline significance [summary RR: 1.49; 95% CI, 0.99–2.25, p = 0.068; based on 4 studies]. In addition, the magnitude of the reported effect tended to be greater in smaller samples and decreased in samples with a larger number of cases (p_heterogenity_<0.0001) and with a longer duration of follow-up (p_heterogenity_ = 0.001). The effect was slightly stronger in studies measuring ADMA in plasma as compared to studies measuring ADMA in serum (p_heterogenity_ = 0.033). As expected, studies with more comprehensive adjustments displayed weaker associations than studies including fewer confounders (p_heterogenity_ = 0.002).

**Table 2:** Meta-Analysis of ADMA and all-cause mortality or CVD for subgroups.

Factors stratified	summary RR (95% CI)ǂ	No. of studies	P for heterogeneity between subgroups†	summary RR (95% CI)ǂ	No. of studies	P for heterogeneity between subgroups†
	All-cause mortality			CVD		
**All**	1.52 (1.37–1.68)	34		1.33 (1.22–1.45)	30	
**Study population**						
General population	1.30 (1.16–1.47)	5	0.001	1.34 (1.12–1.60)	8	0.170
Patients with renal diseases	1.22 (1.11–1.35)	8		1.17 (1.06–1.29)	9	
Patients with existing CVD	1.67 (1.46–1.90)	16		1.49 (1.30–1.72)	14	
Patients with diabetes	1.49 (0.99–2.25)	4		1.94 (0.80–4.66)	2	
Patients from intensive care unit	4.85 (1.39–16.97)	4		-		
**Number of events**						
<100	2.43 (1.84–3.21)	14	<0.0001	1.35 (1.19–1.53)	16	0.279
100—<200	1.46 (1.23–1.74)	12		1.48 (1.17–1.85)	9	
≥200	1.29 (1.20–1.40)	8		1.23 (1.10–1.37)	5	
Duration of follow-up (by mean)*						
<4.7 years	1.65 (1.46–1.87)	18	0.001	1.37 (1.18–1.59)	13	0.640
≥4.7 years	1.27 (1.16–1.40)	13		1.31 (1.17–1.47)	16	
**Blood sample**						
Plasma	1.64 (1.42–1.88)	24	0.033	1.29 (1.15–1.44)	21	0.466
Serum	1.35 (1.21–1.51)	10		1.37 (1.22–1.54)	10	
**Method**						
HPLC	1.45 (1.27–1.65)	16	0.962	1.34 (1.20–1.50)	18	0.457
Tandem mass spectrometry	1.47 (1.28–1.69)	11		1.25 (1.10–1.41)	8	
ELISA	1.49 (1.30–1.72)	7		1.52 (1.11–2.08)	4	
**Adjustment for important confounders**						
0–2	2.21 (1.70–2.87)	12	0.002	1.25 (1.07–1.46)	6	0.260
3–5	1.41 (1.21–1.65)	8		1.60 (1.24–2.06)	7	
≥6	1.34 (1.20–1.49)	14		1.31 (1.17–1.46)	17	

CI, confidence interval; CVD, cardiovascular disease; HPLC, High-performance liquid chromatography; HR, hazard ratio; ELISA, Enzyme Linked Immunosorbent Assay.

^ǂ^ summary RRs are derived from the maximally adjusted models.

^†^ P for heterogeneity between subgroups was evaluated by meta-regression analysis.

* for all-cause mortality for three studies and for CVD for one study time of follow-up was not available.

The dose-response meta-analysis revealed that the relative risk for all-cause mortality increased by 7% per each 0.1 μmol/L increment of ADMA [summary RR: 1.07, 95% CI, 1.05–1.10, p<0.0001; based on 15 studies]. However, there was evidence for a non-linear association between ADMA and all-cause mortality (p for nonlinearity = 0.035, based on 7 studies; Fig 1b). In a sensitivity analysis, excluding older studies which determined ADMA by HPLC, there was no indication for a non-linear relation (p for nonlinearity = 0.184, based on 3 studies).

### Association between ADMA and CVD

**Fig 2:**
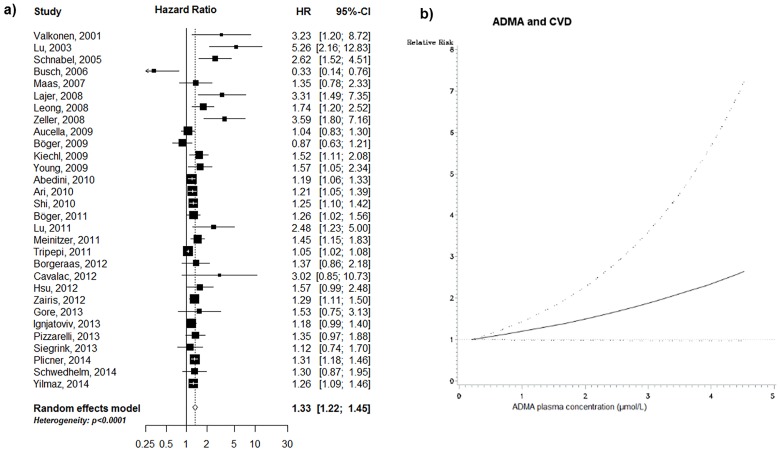
ADMA and CVD: a) high versus low analysis, and b) non-linear dose-response analysis (based on 10 studies, p for non-linearity = 0.370). Black square: point estimate for individual study; horizontal line: 95% CI for observed effect in each study; diamond: pooled estimate and 95% CI for meta-analysis. Random-effects estimate (DerSimonian and Laird method).

We observed a positive association of ADMA levels with incident CVD, based on 30 studies with 30,624 subjects and 3,396 incident CVD events; mean follow-up: 6.0 years). Individuals in the top ADMA tertile had 33% greater risk of CVD as compared to individuals in the bottom tertile (summary RR; 1.33; 95% CI, 1.22–1.45, p<0.0001; Fig 2a). Statistically significant heterogeneity across studies was observed (I^2^ = 76.6%, p<0.0001).

In stratified analyses, the positive association between ADMA and CVD could be observed in all relevant subgroups, e.g. in the general population and all clinical samples, in larger and smaller studies, and in studies with longer and shorter follow-up periods (Table 2).

Modeled as a continuous trait, we observed a 5% increase in CVD risk per each 0.1 μmol/L increment in ADMA [summary RR (95% CI): 1.05 (1.03–1.06), p<0.0001; including 19 studies]. There was no evidence for a non-linear relation between ADMA and CVD (p for non-linearity = 0.370, based on 10 studies; Fig 2b). Excluding studies which determined ADMA with HPLC, results remained unchanged (p for non-linearity = 0.992, based on 10 studies).

### Association between SDMA and all-cause mortality

**Fig 3:**
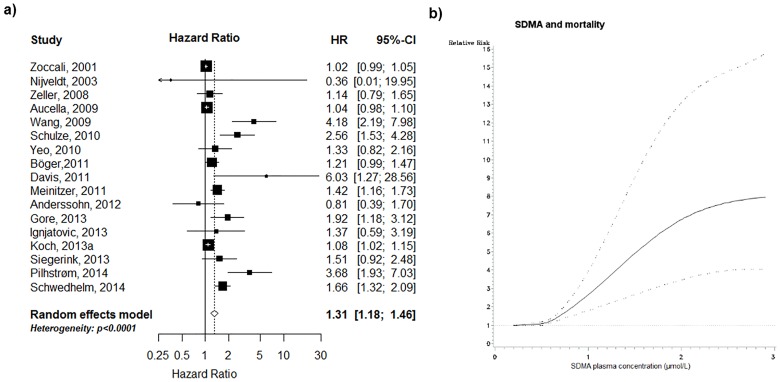
SDMA and all-cause mortality: a) high versus low analysis, and b) non-linear dose-response analysis (based on 4 studies, p for non-linearity = 0.010). Black square: point estimate for individual study; horizontal line: 95% CI for observed effect in each study; diamond: pooled estimate and 95% CI for meta-analysis. Random-effects estimate (DerSimonian and Laird method).

In our meta-analysis, based on 17 studies and (18,163 individuals, 2,903 deaths; mean follow-up 4.6 years) SDMA was associated with increased risk of all-cause mortality in the top as compared to the bottom SDMA tertile [(summary RR (95% CI): 1.31 (1.18–1.46), p<0.0001; Fig 3a]. There was evidence for statistically significant heterogeneity between studies (I^2^ = 82.1%, p<0.0001). In subgroups analyses (Table 3), the association was stronger in the general population as compared to defined clinical settings, (p_heterogeneity_ = 0.003 across all samples), and more prominent in studies with a larger number of events (p_heterogeneity_ = 0.009) and longer duration of follow-up (p_heterogeneity_ = 0.004). We also observed slight differences in the strength (but not in the direction) of the association depending on the method of SDMA assessment (p_heterogeneity_ = 0.020). As expected, studies with a parsimonious set of confounders reported stronger associations as compared to full-adjusted analyses (p_heterogeneity_ = 0.055).

**Table 3:** Meta-Analysis of SDMA and all-cause mortality or CVD for subgroups.

Factors stratified	summary RR (95% CI)ǂ	No. of studies	P for heterogeneity between subgroups†	summary RR (95% CI)ǂ	No. of studies	P for heterogeneity between subgroups†
	All-cause mortality			CVD		
**All**	1.31 (1.18–1.46)	17		1.36 (1.10–1.68)	13	
**Study population**						
General population	1.71 (1.39–2.10)	2	0.003	2.00 (1.42–2.82)	3	0.023
Patients with renal diseases	1.12 (0.99–1.25)	5		1.12 (0.88–1.41)	6	
Patients with existing CVD	1.52 (1.15–2.02)	7		1.27 (0.79–2.05)	5	
Patients with diabetes	-	-		-	-	
Patients from intensive care unit^§^	1.28 (0.84–1.94)	4		-		
**Number of events**						
<100	1.14 (0.92–1.42)	6	0.009	1.52 (1.08–2.16)	9	0.583
100—<200	1.06 (0.99–1.13)	7		1.51 (1.09–2.09)	2	
≥200	1.52 (1.22–1.90)	4		1.17 (0.76–1.78)	2	
Duration of follow-up (by mean)*						
<4.7 years	1.13 (1.02–1.25)	9	0.004	1.19 (0.83–1.70)	6	0.297
≥4.7 years	1.59 (1.29–1.96)	6		1.52 (1.14–2.03)	7	
**Blood sample**						
Plasma	1.27 (1.11–1.46)	12	0.369	1.23 (0.97–1.56)	10	0.134
Serum	1.46 (1.11–1.91)	5		1.56 (1.28–1.91)	3	
**Method**						
HPLC	1.18 (1.04–1.33)	9	0.020	1.31 (0.98–1.76)	8	0.563
Tandem mass spectrometry	1.72 (1.28–2.30)	7		1.52 (1.01–2.29)	5	
**Adjustment for important confounders**						
0–2	2.26 (1.28–3.99)	7	0.055	5.10 (0.96–27.15)	2	0.214
3–5	1.32 (0.97–1.78)	4		1.31 (0.57–3.04)	4	
≥6	1.15 (1.04–1.27)	6		1.15 (0.96–1.37)	7	

CI, confidence interval; CVD, cardiovascular disease; HPLC, High-performance liquid chromatography; HR, hazard ratio.

^ǂ^ summary RRs are derived from the maximally adjusted models.

^†^ P for heterogeneity between subgroups was evaluated by meta-regression analysis.

^§^ only one study investigated SDMA and all-cause mortality or CVD, respectively in patients with diabetes.

* for all-cause mortality for two studies time of follow-up was not available.

The dose-response meta-analysis for all-cause mortality revealed in a 4% risk increase (95% CI: 2%-5%) per 0.1 μmol/L increment of SDMA (p<0.0001, based on 10 studies). However, there was also an indication for a non-linear association between SDMA and all-cause mortality (p for non-linearity = 0.010, based on 4 studies; Fig 3b). When excluding studies HPLC-based measurements, there was no evidence for non-linearity (p = 0.425). However, this analysis was based on 2 studies only.

### Association between SDMA and CVD

**Fig 4:**
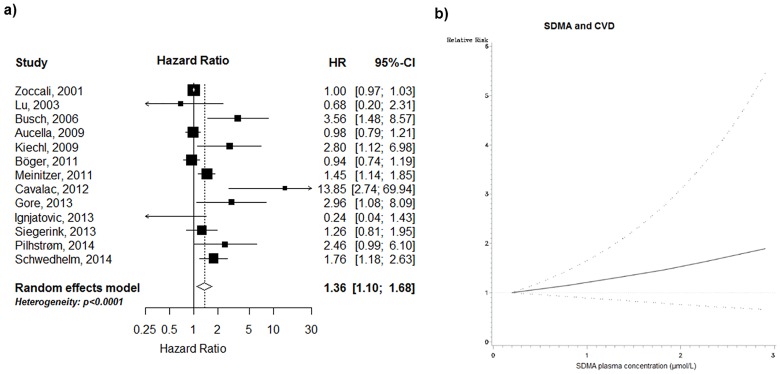
SDMA and CVD: a) high versus low analysis, and b) non-linear dose-response analysis (based on 5 studies, p for non-linearity = 0.059). Black square: point estimate for individual study; horizontal line: 95% CI for observed effect in each study; diamond: pooled estimate and 95% CI for meta-analysis. Random-effects estimate (DerSimonian and Laird method).

The risk for incident CVD (based on 13 studies, including 16,807 subjects and 1,534 cases; mean follow-up 4.9 years) was 36% higher for participants in the upper tertile compared to participants in the lowest tertile [summary RR (95% CI): 1.36 (1.10–1.68); Fig 4a). Statistically significant heterogeneity between studies was observed (I^2^ = 76.6%; p<0.0001). In subgroup analyses, the association was strongest in samples from the general population (as compared to clinical settings; p_heterogeneity_ = 0.023), but no major differences between the other subgroups evaluated could be observed (Table 3).

In the dose-response meta-analysis, the risk of CVD increased by 3% per 0.1 μmol/L increment of SDMA [borderline significant: summary RR (95% CI): 1.03 (1.00–1.06), p<0.072; based on 9 studies]. There was no evidence for a non-linear relation between SDMA and CVD (p for non-linearity = 0.059, based on 5 studies; Fig 4b). Consistently, there was no indication for a non-linear trend after excluding of studies using HPLC for biomarker measurements (p for non-linearity = 0.096, based on 3 studies).

## Discussion

The main results of our systematic review and meta-analysis were as follows. First, circulating concentrations of both biomarkers, ADMA and SDMA, were independently associated with a modestly increased risk of all-cause mortality and of incident CVD. Second, the association of ADMA with CVD was robust in all examined subgroups and across all study populations; its association with mortality was likewise observed in many subgroups but particularly strong in critically ill individuals. Third, the association with SDMA and both outcomes, mortality and CVD, was particularly strong in samples from the general population.

### In the context of the published literature

#### ADMA and all-cause mortality

Individuals with ADMA levels in the top tertile had an about 50% increased risk of all-cause mortality compared with individuals in the bottom tertile of ADMA levels and was particularly strong in critically ill patients on intensive care units. In the literature, the most profound elevations of ADMA have been reported in patients with organ failure such as seen in sepsis. Thus the extent of elevation of ADMA in critically ill patients may simply reflect the severity of organ failure,[34] which in turn is strongly related to adverse outcomes. In addition, based on excess mortality observed in clinical trials of NOS-inhibitors in critically ill patients,[71] it has been speculated that ADMA may also aggravate clinical outcomes.[72] Recent data from mice with impaired ADMA metabolism point in the same direction.[73]

As expected, slight differences between studies were observed, with slightly smaller effects being reported in studies of higher quality (higher number of events, longer duration of follow-up and higher number of included confounders in the statistical analysis). In addition, associations were slightly stronger for studies that measured ADMA in plasma compared to studies measuring it in the serum. In our dose-response meta-analysis there was evidence for a non-linear relation between ADMA and all-cause mortality, with a steeper increase in risk for ADMA levels around >0.6 μmol/l; however, with ADMA concentrations around >3.0 μmol/l the risk slightly decreased. In a sensitivity analysis, restricted to studies that assessed ADMA by tandem mass spectrometry and ELISA, the association between ADMA and all-cause mortality was almost linear.

#### ADMA and CVD

We observed a relative CVD risk increase of 33% in individuals in the top vs. the bottom ADMA tertile. Our results are consistent with a previous, slightly smaller meta-analysis based on 22 prospective studies, including 19,842 subjects and 2,339 CVD events. Indeed, Willeit and colleagues reported a very similar summary effect estimate [RR (95%CI): 1.42 (1.29–1.56)]. Exclusion criteria of the latter meta-analysis were stricter compared to our criteria, and thus our meta-analysis was based on more studies and a higher number of individuals and events (30 studies, 30,624 subjects and 3,396 CVD cases). Our results are consistent with some prior analyses in individual studies and we demonstrate that after including all currently available studies we see a positive association between ADMA and CVD, however, some individual studies, including the Framingham study, failed to observe an association between ADMA and CVD.

#### SDMA and all-cause mortality

Our meta-analysis indicated that individuals in the top SDMA tertile were at higher risk (RR: 31%) of dying from any cause as compared to individuals in the bottom SDMA tertile. The association was strongest in samples from the general population and less prominent in patients with prevalent cardiovascular disease. In patients with renal failure, the association failed to reach statistical significance. SDMA is much more sensitive to changes in renal function as compared to ADMA,[74] which may explain its weaker independent association with adverse outcomes in renal disease when renal function is adjusted for. Similar to the findings regarding ADMA and all-cause mortality, the strongest increase of risk was observed at SDMA levels approximately >0.6 μmol/l, and with extreme values, the risk increase was attenuated. However, excluding HPLC-based SDMA measurement, there was no indication for a non-linear association.

#### SDMA and CVD

In our meta-analysis, SDMA was associated with 36% (95% CI: 10% to 68%) increased risk of CVD, by comparing individuals in the top tertile with individuals in the bottom tertile of SDMA. In the meta-analysis mentioned above, a very similar risk estimate was reported that failed to reach statistical significance, [summary RR (95% CI) 1.32 (0.92–1.90)], probably due to the smaller number of studies (n = 8; total n = 9,070) and events (n = 848) included.[30] In our report, we included 13 studies with a total of 16,807 individuals (events = 1,534). Our findings were consistent in analyses stratified by study characteristics, method of ADMA assessment and duration of follow-up. However, as in our analyses relating SDMA to all-cause mortality, the association with CVD risk was likewise strongest in samples from the general population.

### Change in methods of biomarker determination over time

Stratification of studies by the methodology used to determine ADMA/SDMA showed some degree of heterogeneity with respect to total mortality which is unlikely to be attributable to clinical differences between the underlying studies.

As with any new biomarker, the methodology for the quantification of ADMA and SDMA has advanced over recent years and has become more and more standardized.[75] Initial clinical studies predominantly relied on high pressure liquid chromatography (HPLC) methods for the detection of ADMA and SDMA.[3, 7, 8] In these early studies, biomarker quantification—especially in absolute terms—was rather challenging due to the limited availability of defined pure ADMA and SDMA standards and reference samples. Limited knowledge regarding the prevalence and interference by chemically related compounds added further uncertainties. This may explain the continuous decline in the mean ADMA plasma concentration reported for similar clinical settings, going down from 0.95 μmol/l for healthy controls in 2001 to 0.41 μmol/l in 2011.[7, 76] In the case of SDMA, matters may have been further aggravated by the fact that it was considered to be a biologically inactive byproduct and thus may have received less methodological scrutiny. The less well validated methodology for SDMA determination may also have contributed to the initially mostly negative results regarding SDMA as a risk marker. It may also explain why commercial immunoassays emerged much earlier for ADMA than for SDMA.[9, 32] The introduction of mass spectrometric methods allowed the more reliable determination of reference values for ADMA and SDMA, [77–79] which may help to calibrate and (re)interpret early ADMA and SDMA data.

### ADMA and SDMA levels and risk prediction and clinical utility

In our present-meta analysis, we confirm a positive and statistical significant association of ADMA and SDMA with all-cause mortality and CVD. All associations were relatively consistent across multiple subgroups, but varied in magnitude. In order for ADMA or SDMA measurements to be considered applicable for routine clinical use it needs to be demonstrated, that measuring ADMA or SDMA affects patient management and ultimately patient outcome.[80, 81]

Wang et al. suggested possible additive effects of ADMA and SDMA with respect to the predictions of major adverse cardiac events and proposed an “arginine methylation index” ([ADMA+SDMA]/Monomethyl-Arginine).[59] This index was statistically independently associated with MACE. However, only few studies investigated whether the addition of ADMA or SDMA improves established indices of risk prediction models, including improvement in discrimination, calibration, and reclassification.[81] Gore and colleagues reported statistically significant increase in the C-statistic after adding SDMA to a model predicting all-cause mortality and CVD in the general population.[15] However, the absolute increments in discrimination were unlikely to be clinically relevant. Other studies did not find a relevant increment in the C-statistic once ADMA or SDMA were added to the statistical prediction models in different clinical settings.[11, 15, 18, 23, 69] This concept also warrants further investigation. It needs to be established, however, whether inclusion of ADMA or SDMA improves risk prediction models (e. g. discrimination, reclassification)[81] beyond classic risk factors or that the increased risk for CVD and mortality associated with higher ADMA or SDMA can be specifically treated. It can be speculated that in clinical practice determination ADMA may be especially useful in critically ill patients while SDMA may be more suitable and useful in the general population. Further studies are warranted to confirm or refute this hypothesis.

In contrast to other risk markers like LDL-cholesterol distinct pharmacological targeting of ADMA or SDMA has not been clinically successful, so far. Still, assuming a causal role in CVD further approaches have been proposed[82]:

1. Direct targeting of circulating ADMA or SDMA. In principle ADMA or SDMA concentrations could be reduced by a decrease of dietary uptake or endogenous generation of ADMA/SDMA or by increased elimination (i.e. increase of cellular exchange, transport and metabolism).[2]
2. Treatment of putative pathophysiological effects of ADMA or SDMA. In case of ADMA, L-arginine has widely been advocated as an "antidote".[83] However, supplementation of L-arginine irrespective of underlying ADMA-levels failed to improve cardiovascular outcome and prospective studies with long term supplementation of L-arginine in patients with elevation of ADMA and follow-up for relevant clinical endpoints (mortality) remain to be conducted.[84]

### Shared and distinct properties of ADMA and SDMA

ADMA and SDMA are chemically closely related and both associated with incident CVD and total mortality. However, the present study indicates that both compounds may slightly differ in the pattern and in the strength of their association with clinical endpoints within the same cohort as well as across distinct clinical settings.

Methodological aspects, discussed further above, aside these observations are not surprising, considering the overlapping as well as distinct metabolic and (patho-) physiological properties of ADMA and SDMA (summarized in Table 4). It also is possible, if not likely, that ADMA and SDMA may act both as risk markers as well as risk factors. The plasma levels of ADMA and SDMA are affected by several overlapping as well as distinct metabolic pathways.[3, 4, 85–90] Elevation of ADMA may predominantly represent impaired dimethylarginine dimethylaminohydrolase (DDAH) activity [4] while elevation of SDMA more likely reflects impaired renal function [91, 92] and/or impaired alanine—glyoxylate aminotransferase 2 (AGXT2) activity (i.e. hyper beta-aminoisobutyric acid uria).[89, 93, 94] Moreover, ADMA and SDMA have overlapping as well as distinct biological effects (Table 4). While both compounds may alter cellular exchange (i.e. transport) of L-arginine,[1] only ADMA appears to be a direct inhibitor of NOS.[2, 3] Independent of the L-arginine-NO-pathway SDMA may exercise biological effects through alternative mechanisms augmenting oxidative stress or activate toll-like receptors.[95, 96]

**Table 4:** Differences and similarities in the biological properties of ADMA and SDMA.

	ADMA	SDMA
Generation / source [2]	**Endogenous formation** [3] Monomethylation of protein-bound L-arginine by type I and II PRMTs [85] Asymmetric dimethylation of protein-bound monomethylarginine by type I PRMTs [85] Liberation of ADMA by protein degradation [86]	**Endogenous formation** [3] Monomethylation of protein- bound L-arginine by type I and II PRMTs [85] Asymmetric dimethylation of protein-bound monomethylarginine by type II PRMTs [85] Liberation of SDMA by protein degradation [87]
	**Exogenous/ dietary uptake** Exact contribution unknown	**Exogenous/ dietary uptake** Exact contribution unknown
Distribution / transport [88]	Cellular uptake and efflux mediated by cationic amino acid transporters	Cellular uptake and efflux mediated by cationic amino acid transporters
Elimination [2]	**Metabolism** Major route of elimination [3, 89, 90] Major metabolising enzymes DDAH1 and DDAH2 [4] AGXT2 [91] Butylation and Methylation [90]	**Metabolism** Minor route of elimination [89, 90] No substrate of DDAHs AGXT2 [91] (Major metabolising enzyme) Butylation and Methylation [90, 94]
	**Renal Excretion** Minor route of elimination [92, 93]	**Renal Excretion** Major route of elimination [92, 93]
Biological effects	Inhibition of nitric oxide synthases (eNOS, nNOS and iNOS) [2, 3] Weak inhibition of L-arginine transport [88] Activation of NF-κB with enhanced expression of inflammatory cytokines [95]	No clinically relevant direct inhibition of nitric oxide synthases [3] Possible weak indirect inhibition of NO- Synthesis [96] Weak inhibition of L-arginine transport [88] Activation of NF-κB with enhanced expression of inflammatory cytokines [97] Increase in monocytic ROS production by enhanced activation of store-operated Ca2+- channels [98] Modification of HDL activating toll like Receptors [99]

ADMA, Asymmetric dimethylarginine; AGXT2; alanine—glyoxylate aminotransferase 2; DDAH, dimethylarginine dimethylaminohydrolase; eNOS, endothelial nitric-oxide synthase; HDL, high density lipoprotein; iNOS, inducible nitric-oxide synthase; NF-κB, nuclear factor kappa-light-chain-enhancer of activated B cells; nNOS; neuronal nitric oxide synthase; PRMT, protein arginine N-methyltransferase; ROS, reactive oxygen species; SDMA, symmetrical dimethylarginine

### Strengths and limitations

Our meta-analysis has several strengths. To our knowledge, there is no prior large scale meta-analysis quantifying the association between ADMA, SDMA and all-cause mortality. Regarding the association of both biomarkers with CVD, the present study includes a much larger number of studies, patients and clinical events than any previous review or meta-analysis for ADMA or SDMA. This allowed us to consider key methodological factors and subgroup analyses. In addition, we also performed a dose-response meta-analysis of the relation between ADMA; SDMA and all-cause mortality or CVD and assessed the exact shape of the association between biomarker and outcome.

However, our meta-analysis has some limitations that merit consideration. First, we observed high heterogeneity between studies included in our meta-analysis. Thus, we performed stratified analyses by accounting for different study populations, study characteristics and assessment methods of ADMA and SDMA. Slight differences between subgroups were detected, but in general, effects were present across all or most strata. Second, regarding the dose-response analysis, the number of studies we could include was limited because of partially incomplete information in individual studies. Thus, we were not able to stratify the dose-response meta-analysis by different populations or methodological approaches, and more research exploring dose-response relations is warranted. Third, the majority of the included studies were based on diseased cohorts and evidence of the association between particularly SDMA and all-cause mortality or CVD in the community is still rare. Thus, more population-based studies investigating this association are needed. Fourth, we conducted our literature search in Medline only and thus, it might be possible that we missed few of the relevant studies. However, we hand-searched all included studies and reviews to check for additional relevant articles.

## Conclusions

In conclusion, this meta-analysis including a large number of prospective studies indicates that ADMA and SDMA are independently associated with all-cause mortality and CVD across a broad spectrum of populations and clinical conditions.

## Supporting Information

S1 Fig PRISMA checklist. (PDF)

S2 Fig Flowchart of study selection for the meta-analysis. (PDF)

S1 Table Considered confounders of each study included in the meta-analysis. (PDF)

## Abbreviations

ADMA: Asymmetric dimethylarginine
AGXT2: alanine—glyoxylate aminotransferase 2
CVD: Cardiovascular disease
DDAH: dimethylarginine dimethylaminohydrolase
ELISA: Enzyme Linked Immunosorbent Assay
eNOS: endothelial nitric-oxide synthase
F: female
HDL: high density lipoprotein
HPLC: High-performance liquid chromatography
HR: Hazard ratio
iNOS: inducible nitric-oxide synthase
M: male
MACE: major adverse cardiac event
NF-κB: nuclear factor kappa-light-chain-enhancer of activated B cells
nNOS: neuronal nitric oxide synthase
NO: Nitric oxide
OR: Odds ratio
PRMT: protein arginine N-methyltransferase
ROS: reactive oxygen species
RR: Relative risk
SD: Standard deviation
SDMA: Symmetric dimethylarginine
