# Supplementary figures and images for: Asymmetric and Symmetric Dimethylarginine as Risk Markers for Total Mortality and Cardiovascular Outcomes: A Systematic Review and Meta-Analysis of Prospective Studies
Source: PLoS One. 2016 Nov 3;11(11):e0165811. doi: 10.1371/journal.pone.0165811 (PMC5094762; doi:10.1371/journal.pone.0165811)

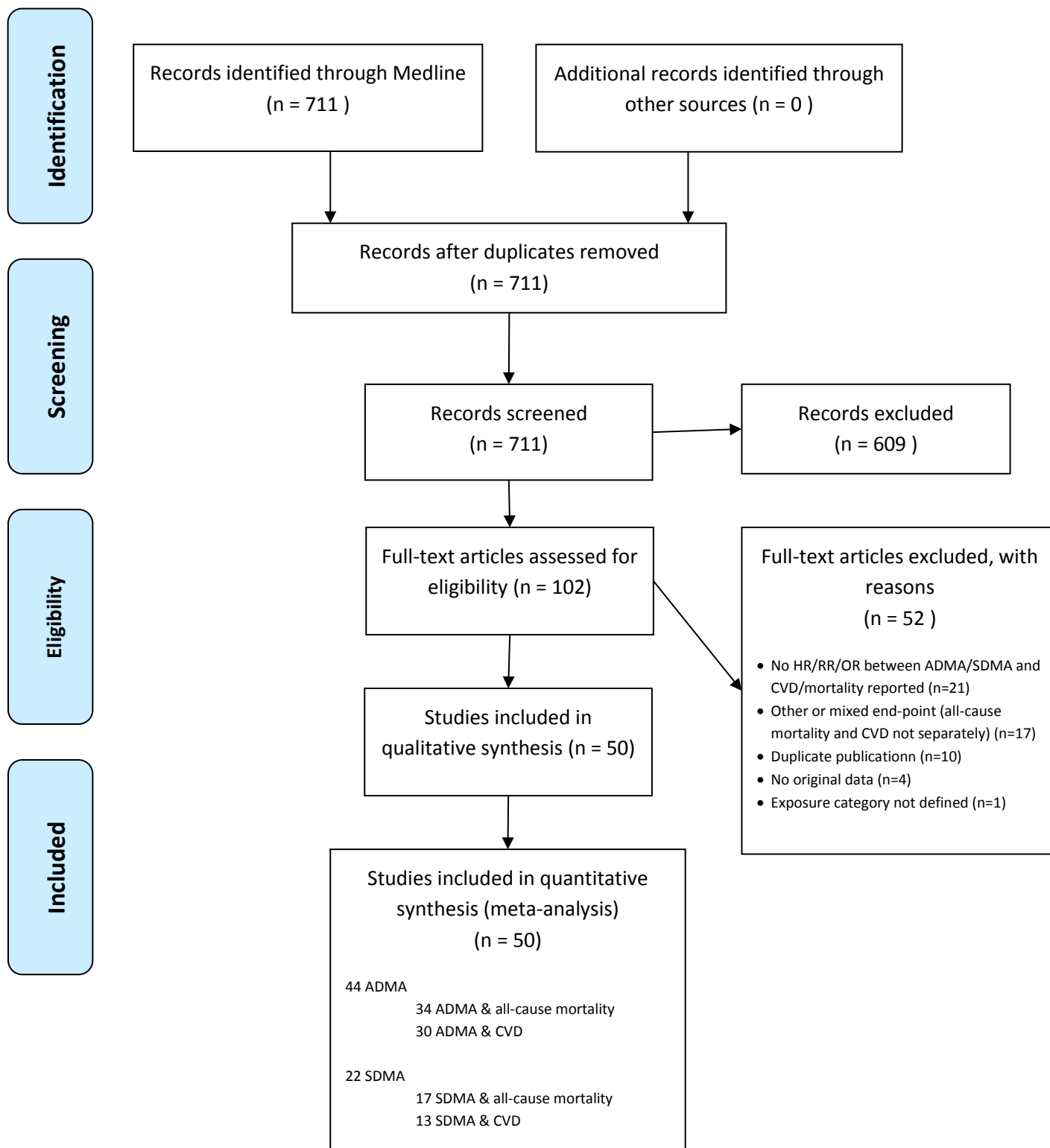

**S2 Figure:** Flowchart of study selection for the meta-analysis

Supplement: S2 Fig — (PDF) [file pone.0165811.s002.pdf]
