# Supplementary material for: Asymmetric and Symmetric Dimethylarginine as Risk Markers for Total Mortality and Cardiovascular Outcomes: A Systematic Review and Meta-Analysis of Prospective Studies
Source: PLoS One. 2016 Nov 3;11(11):e0165811. doi: 10.1371/journal.pone.0165811 (PMC5094762; doi:10.1371/journal.pone.0165811)
Supplement: S1 Table — (PDF) [file pone.0165811.s003.pdf]

S1 Table: Considered confounders of each study included in the meta-analysis

Study	Confounders										
	Age	Sex	BMI or waist	Smoking	CV history	Diabetes	Blood pressure/ hypertension	Blood lipids/ hyperlipidemia	Family history of CVD	GFR/ eGFR	Other
Valkonen et al., 2001		na				✓	✓	✓	✓		
Zoccali et al., 2001	✓	✓		✓	✓	✓	✓	✓			L-arginine, SDMA, fibrinogen, CRP, homocysteine, haemoglobin, calcium phosphate product, albumin, duration of dialysis treatment, fractional urea clearance
Lu et al., 2003				✓	✓	✓		✓			final minimal luminal diameter, acute gain, L-arginine, L-arginine/ADMA-ratio, ADMA or SDMA
Nijveldt et al., 2003	Not reported										
Ravani et al., 2005										✓	Not all reported: proteinuria, hemoglobin, homocysteine
Schnabel et al., 2005	✓	✓	✓	✓	✓	✓	✓	✓		✓	Angiotensin-convertig enzyme inhibitor, statin, ß-Blocker, extent of vessel disease; BNP, CRP
Busch et al., 2006 (unadjusted for SDMA)	✓		✓		✓	✓					CRP, heamodialysis, haemoglobin, creatinine, SDMA, albumin
Mittermayer et al., 2006	✓	✓	✓	✓	✓	✓	✓	✓			Statin, hsCRP, creatinine
Maas et al., 2007	✓	na	✓	✓			✓	✓		✓	Survey, education, alcohol, physical activity
Nicholls et al., 2007	✓		✓				✓				Creatinine clearance
Skoro-Sajer et al., 2007	✓	✓									cardiac index, pulmonary arterial pressure, right atrial pressure, pulmonary vascular resistance, L-arginine
Lajer et al., 2008 (only for CVD)	✓	✓		✓	✓	✓	✓	✓	✓	✓	Antihypertensive treatment
Leong et al, 2008	✓	na	✓	✓		✓	✓	✓			Cohort, creatinine clearance, homocysteine, vitamin B12
Wilson Tang et al., 2008	✓									✓	Left ventricular ejection fraction
Zeller et al., 2008 (only for all-cause mortality)	✓						✓			✓	NT-proBNP, GRACE risk score, CRP, homocysteine, LVEF peak CK, statin
Aucella et al., 2009 (only for ADMA and all-cause mortality)	✓	✓		✓	✓	✓	✓	✓			Albumin, Dialysis modality, RDT duration, Hb, albumin, homocysteine, antihypertensive treatment, treatment with erythropoietin, SDMA L-arginine
Böger et al., 2009	✓	✓		✓	✓ (only for all-cause mortality)	✓	✓	✓			Antihypertensive treatment, creatinine, BNP, UACR; for all-cause mortality also for: rennin, homocysteine, CRP
Cavusoglu et al., 2009	✓	na			✓						Creatinine, left ventricular function, number of diseased coronary arteries, hs-CRP, fibrinogen,
Kiechl et al., 2009	✓	✓	✓	✓	✓	✓	✓	✓			Social status, heart failure, physical activity, homocysteine, osteoprotegerin, soluble RANKL. GF-MDRD, urinary albumin, CRP
Wang et al., 2009											
Young et al., 2009	✓	✓	✓	✓	✓	✓	✓	✓		✓	Race, randomization assignments, CRP, proteinuria, cause of kidney disease

Abedini et al., 2010	✓	✓		✓	✓	✓	✓	✓		Creatinine
Ari et al., 2010										Hs-CRP
Cavusoglu et al. 2010	✓	na								Creatinine, no. of diseased coronary arteries, fibrinogen
Schulze et al., 2010	✓				✓					Stroke subtype, AF
Shi et al., 2010	✓			✓	✓		✓	✓	✓	Pre-albumin, calcium, intact PTH, uric acid, LVDs, LVPWT, LAD, LVST, LVMI, EF
Yeo, et al., 2010 (only for ADMA)										Creatinine, Angiopoeitin 2, HRP2
Böger, et al., 2011	✓	✓	✓	✓	✓	✓			✓	PAD, homocysteine, hs-CRP
Davis et al., 2011										
Lu et al., 2011b	✓	✓		✓	✓	✓	✓	✓	✓	Acute coronary syndrome
Meinitzer et al., 2011	✓	✓	✓	✓	✓		✓	✓	✓	
Tripepi et al., 2011	✓	✓			✓	✓	✓	✓		Antihypertensive treatment, dialysis vintage, albumin, hemoglobin, calcium, phosphate, homocysteine
Anderssohn et al., 2012 (only for SDMA)	✓	✓					✓		✓	NT-proBNP, NYHA functional class, use of ß-blockers
Borgeraas et al., 2012	✓	✓		✓	✓	✓	✓			Left ventricular ejection fraction
Cavalca et al., 2012	✓									Left ventricular ejection fraction, hemoglobin
Hsu et al., 2012	✓								✓	Left ventricular ejection fraction, NT-proBNP
Visser et al., 2012										
Zairis et al., 2012	✓	✓					✓		✓	Heart rate, atrial fibrillation, acute pulmonary oedema, left ventricular ejection fraction, natrium, brain natriuretic peptide
Gore et al., 2013	✓	✓	✓	✓	✓	✓	✓	✓	✓	Race, hs-CRP, pro–brain-type natriuretic peptide, cardiac troponin T
Ignjatovic et al., 2013	✓	✓		✓		✓	✓			
Koch et al., 2013a										Creatinine, bilirubin, white blood cell count, lactate
Koch et al., 2013b										Creatinine, bilirubin, white blood cell count, lactate
Pizzarelli et al., 2013	✓		✓	✓			✓	✓		Homocysteine, albuminuria, anti-hypertensives, hemoglobin, l-arginine, calcium
Siegerink et al., 2013	✓	✓	✓	✓	✓	✓	✓	✓	✓	CRP, discharge prescription of diuretics, ACE inhibitors, CKD-EPI
Drew et al., 2014	✓	✓			✓	✓				Dialysis vintage, vascular access type, history of congestive heart failure
Levin et al., 2014	✓	✓			✓	✓			✓	HF, phosphate, albumin, , NT-Pro-BNP, hs-CRP, FGF23
Pihlstrøm, 2014										
Plicner et al., 2014										
Schwedhelm et al., 2014	✓	✓	✓	✓						Physical activity
Yilmaz et al., 2014	✓									Flow-mediated dilatation, pentraxin 3
